# Reply: Clinical trials on onabotulinumtoxinA for the treatment of chronic migraine

**DOI:** 10.1007/s10194-011-0334-4

**Published:** 2011-04-03

**Authors:** Sheena Aurora, Hans-Christoph Diener, David Dodick

**Affiliations:** 1 Swedish Neuroscience Institute, Seattle, WA USA; 2 University of Essen, Essen, Germany; 3 Mayo Clinic Arizona, Phoenix, AZ USA

Dr. Russell raises important points regarding the diagnostic criteria for chronic migraine (CM) and medication overuse headache (MOH). We agree that the definitions for these entities have posed challenges for the past two decades. The ICHD-2 diagnostic criteria for CM and MOH were evolving as the PREEMPT clinical trial program was developed and launched. In the original ICHD-2 definition of MOH, remission of headache >15 days per month following discontinuation of medication was required. As a consequence, the diagnosis could be assigned only to individuals who no longer had the condition. The ICHD-2R definition of MOH no longer requires remission after withdrawal [1]. A history of headache escalation during a period of medication overuse is still required by ICHD-2R, though in the vast majority of patients it is not possible to reliably determine if medication overuse is a cause or a consequence of increasing headache frequency. Moreover, contrary to conventional wisdom, there is no evidence from controlled trials that the withdrawal of acute medications alone, in those who “overuse” them, leads to the long-term remission of headache.

In the PREEMPT program, patients who otherwise met study criteria for CM were not excluded if they were making frequent use of acute medication. Contrary to Dr. Russell’s assertion, additional data are needed to determine which of these patients meet criteria for MOH. The decision not to exclude patients overusing acute medication was made based on consultation with members of the Task Force of the International Headache Society Clinical Trials Subcommittee, and is consistent with their published guidelines for controlled trials of prophylactic treatment of CM in adults [2]. These guidelines reflect the high prevalence of medication overuse in CM patients, and recommend the inclusion and stratification of these patients in clinical studies. In line with these guidelines, patients with medication overuse were stratified at randomization in the PREEMPT clinical program [3]. If patients with medication overuse are excluded, we lose the opportunity to address the benefits of treatment in a large group with disabling headache and an unmet treatment need. Results from an independent clinic-based study designed to assess the overlap between ICHD-2R and other proposed diagnostic criteria for CM (including PREEMPT enrollment criteria) determined that there was significant overlap between these definitions [4].

We completely agree with Dr. Russell that subgroup analyses are warranted. Data have been presented on the medication overuse subpopulation demonstrating efficacy of onabotulinumtoxinA compared to placebo on multiple headache symptom measures [5], and a manuscript is in progress. Additional post hoc analysis of subgroup populations, such as those PREEMPT patients who have taken prior prophylaxis, are currently underway and will also be detailed in future publications.

We believe the PREEMPT study enrolled patients representative of the CM population and that the results of the program have greater generalizability because of our inclusion criteria. These studies show that onabotulinumtoxinA treatment compared to placebo resulted in clinically meaningful outcomes; significantly reduced headache frequency; and significantly improved functioning, vitality, and overall quality of life [3]. As clinicians dedicated to the care of patients with headache, identification and successful prophylactic treatment of this highly disabled patient population is our main objective.

Sincerely,

Sheena Aurora, MD

Hans-Christoph Diener, MD

David Dodick, MD

On behalf of the PREEMPT Chronic Migraine Study Group
